# Bell’s Palsy After 24 Hours of mRNA-1273 SARS-CoV-2 Vaccine

**DOI:** 10.7759/cureus.15935

**Published:** 2021-06-26

**Authors:** Haris Iftikhar, Syeda Mishkaat U Noor, Maarij Masood, Khalid Bashir

**Affiliations:** 1 Emergency Medicine, Hamad Medical Corporation, Doha, QAT

**Keywords:** covid-19, bell’s palsy, interferons, stroke, facial nerve, vaccine

## Abstract

Coronavirus disease 2019 (COVID-19) has become the fastest-spreading pandemic of the 21st century. Various vaccines have been made available via emergency use authorization. Currently, two mRNA vaccines are being offered internationally, BNT162b2 and mRNA-1273. In randomized trials of these vaccines, the incidence of Bell’s palsy in the vaccinated group does not statistically exceed the placebo group. The FDA recommends increased surveillance for Bell’s palsy as a potential side effect with the administration of the vaccines among larger populations globally. There have been a few case reports of Bell’s palsy associated with mRNA vaccines. Type I interferons have been proposed as the potential mechanism linking mRNA COVID-19 vaccines to Bell’s palsy. Here, we report the case of a 36-year-old previously healthy patient who developed symptoms of Bell’s palsy along with left-arm numbness, tingling, and subjective weakness masquerading as a subacute stroke after receiving the second dose of the mRNA-1273 vaccine. CT and MRI of the brain were unremarkable. He was discharged home with a diagnosis of Bell’s palsy and improved on follow-up. mRNA COVID-19 vaccines may be considered a risk factor for Bell’s palsy.

## Introduction

Coronavirus disease 2019 (COVID-19) has become the fastest-spreading pandemic of the 21st century. Globally, the number of cases reached up to 175 million in June 2021 [1]. The global scientific community has been rapidly working toward the prevention of COVID-19. Various vaccines have been made available via emergency use authorization. At present, two mRNA vaccines are being offered internationally, BNT162b2 (Pfizer-BioNTech) and mRNA-1273 (Moderna). No severe safety concerns were identified for either of the vaccines in the randomized clinical trials. There has been an increased propensity of Bell’s palsy cases between the vaccinated group compared to placebo but a causal relationship could not be established [2]. As reported by the two large phase 3 vaccine trials, eight cases of suspected Bell’s palsy were observed: seven from the vaccinated arm and one from the placebo group (this data is derived from the 73,799 volunteers, of which 36,901 received at least one dose of the vaccine) [3]. Specifically, during the mRNA-1273 vaccine, four cases of Bell’s palsy were noted during the observation period of the trial, of which three were from the vaccinated group. For the BNT162b2 vaccine, four vaccinated participants were noted to develop Bell’s palsy [2,3]. In randomized trials, the incidence of Bell’s palsy in the vaccinated group does not statistically exceed the expected frequency in the general population. The FDA recommends increased surveillance for Bell’s palsy as a potential side effect with the administration of the vaccines among larger populations globally [4].

A few case reports of Bell’s palsy associated with mRNA vaccines have been published after the initial randomized trials. According to our literature search, until June 2021, there is only one reported case of Bell’s palsy after receiving the first dose of the mRNA-1273 vaccine [5]. Here, we report the case of a 36-year-old previously healthy patient who developed symptoms of Bell’s palsy along with left-arm numbness, tingling, and subjective weakness masquerading as a subacute stroke after receiving the second dose of the mRNA-1273 vaccine.

## Case presentation

A 36-year-old gentleman came to our emergency department (ED) complaining of weakness over the left side of his face. He had received the second dose of the mRNA-1273 severe acute respiratory syndrome coronavirus 2 vaccine one day before the onset of his symptoms in his left deltoid muscle. His symptoms had been ongoing and progressive for the past three days. He mentioned slight difficulty in speaking and eating. He also had mild numbness and tingling of the left arm along with subjective weakness of the left upper limb. He had never experienced such symptoms earlier. There was no prior history of any medical illnesses. No preceding fever or upper respiratory symptoms were present. He denied ear pain, increased sound sensitivity, or a change in taste sensation.

The patient was alert and oriented at the time of examination. He was speaking in full sentences and no slurring of speech was noted. Initial vitals were a heart rate of 66 beats per minute, a temperature of 37°C, maintaining oxygen saturation of 97% on room air, and blood pressure of 120/90 mmHg. Cranial nerve examination showed isolated VII nerve palsy of lower motor neuron type. The patient was unable to close his left eye completely. There was flattening of wrinkle lines above the left eyebrow and drooping of the left corner of the mouth. There was no objective weakness in all four limbs. He had slightly decreased sensations on his left upper limb which spared the lower limbs. His ear examination was normal bilaterally with no vesicles or scabbing and no parotid swelling in front of the ears. The rest of his physical examination was unremarkable.

**Figure 1:**
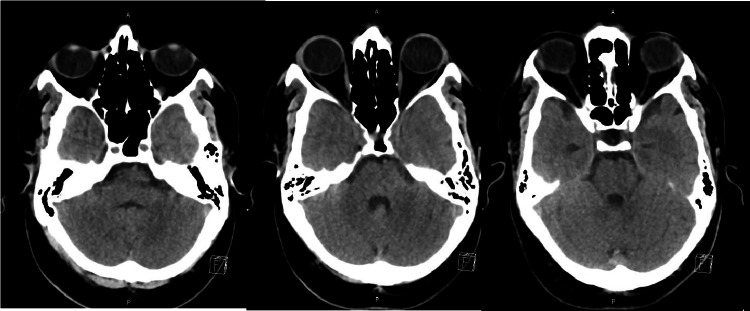
Axial noncontrast CT head of the patient at the level of pons/posterior fossa (multiple sections) showing no abnormalities. CT: computed tomography

**Figure 2:**
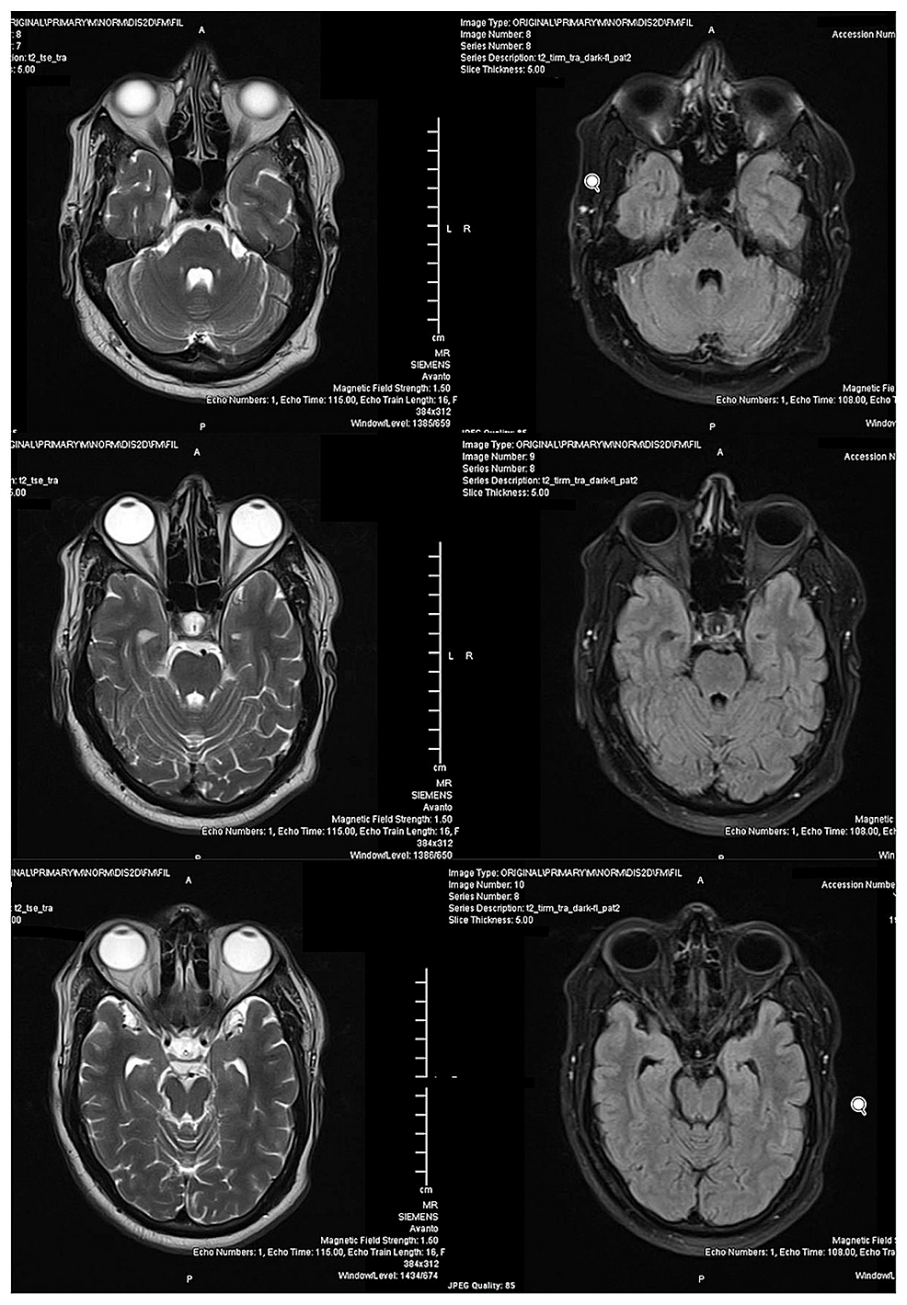
MRI brain of the patient with T2-weighted images (left) and FLAIR images (right) at the level of the pons showing no abnormality. MRI: magnetic resonance imaging; FLAIR: fluid-attenuated inversion recovery

A neurology consult was obtained in the ED due to his left upper limb numbness and weakness along with facial palsy to rule out brainstem stroke. CT and MRI of the brain were done which were unremarkable (Figures 1, 2). A diagnosis of Bell’s palsy was made, and the patient was discharged home with 60 mg of daily oral prednisolone for one week, artificial tears, eye care instructions to prevent corneal injury, and neurology follow-up. On follow-up after two weeks, the patient’s arm symptoms had resolved completely and his facial symptoms had improved but still present.

## Discussion

Bell’s palsy, also known as idiopathic facial nerve paralysis, is considered to be the most common cause of acute facial paralysis of spontaneous origin. Potential contributors to the development of Bell’s palsy include immune, infective, and ischemic mechanisms; however, the exact cause remains unclear. Reactivation of herpes simplex virus infection centered around the geniculate ganglion is suggested as a possible cause [6].

The occurrence of Bell’s palsy has been linked to vaccine administration in the past. A matched case-control study and case series in 2000-2001 found an increased incidence of Bell’s palsy among the receivers of intranasal inactivated influenza vaccine (with an odds ratio of 84%). This phenomenon was thought to be due to the interaction of heat-labile *Escherichia coli* toxin found in the vaccine with the facial nerve. Adverse reports of Bell’s palsy have also been published after the administration of the meningococcal conjugate vaccine [7].

In the published data regarding the mRNA-1273 vaccine, among the 30,351 volunteers who participated in the trial, three cases of Bell’s palsy were reported in the vaccinated group on days 22, 28, and 32 days after vaccination. One incident occurred in the placebo group 17 days after vaccination. The trial concluded that the slight excess of Bell’s palsy in this trial and the BNT162b2 vaccine trial raised a concern that it may be more than a chance event, and this possibility bears close monitoring [2]. A retrospective study with 455 participants found three cases of Bell’s palsy and one with body tingling in individuals who received the first dose of the BNT162b2 vaccine [8].

Other COVID-19 vaccine trials have also reported Bell’s palsy as an adverse event. The single-dose Ad26.COV2.S vaccine used recombinant, replication-incompetent human adenovirus type 26 (Ad26) vector. In a clinical trial of the Ad26.COV2.S vaccine, three cases of Bell’s palsy were reported in the vaccine group compared with two cases in the placebo group. The observed frequency of Bell’s palsy in the vaccine and control group was consistent with the expected background rate in the general population [9]. Nishizawa et al. reported a case of Bell’s Palsy after receiving the Ad26.COV2.S vaccine [10].

Soeiro et al. proposed type I interferons as the potential mechanism linking mRNA COVID-19 vaccines to Bell’s palsy. Therefore, the mRNA COVID-19 vaccine should be considered as an additional risk factor for Bell's palsy [11].

## Conclusions

Data from vaccine trials of mRNA covid vaccines, BNT162b2 and mRNA-1273, suggest that there is an increased propensity of Bell’s palsy cases in the vaccinated group. However, the causal relationship between the mRNA vaccine and Bell’s palsy development needs to be further investigated. Our case highlights the importance of vaccine history in patients presenting to the emergency department with Bell’s palsy. COVID-19 mRNA vaccines can be considered as an additional possible risk factor in the etiology of Bell’s palsy.

## References

[1] (2021). WHO COVID-19 Dashboard. Geneva: World Health Organization. https://covid19.who.int/.

[2] Baden LR, El Sahly HM, Essink B (2021). Efficacy and safety of the mRNA-1273 SARS-CoV-2 vaccine. N Engl J Med.

[3] Cirillo N (2021). Reported orofacial adverse effects of COVID-19 vaccines: the knowns and the unknowns. J Oral Pathol Med.

[4] Colella G, Orlandi M, Cirillo N (2021). Bell's palsy following COVID-19 vaccination [In Press]. J Neurol.

[5] Martin-Villares C, Vazquez-Feito A, Gonzalez-Gimeno MJ, de la Nogal-Fernandez B (2021). Bell's palsy following a single dose of mRNA SARS-CoV-2 vaccine: a case report [In Press]. J Neurol.

[6] Eviston TJ, Croxson GR, Kennedy PG, Hadlock T, Krishnan AV (2015). Bell's palsy: aetiology, clinical features and multidisciplinary care. J Neurol Neurosurg Psychiatry.

[7] Ozonoff A, Nanishi E, Levy O (2021). Bell's palsy and SARS-CoV-2 vaccines. Lancet Infect Dis.

[8] El-Shitany NA, Harakeh S, Badr-Eldin SM (2021). Minor to moderate side effects of Pfizer-BioNTech COVID-19 vaccine among Saudi residents: a retrospective cross-sectional study. Int J Gen Med.

[9] Sadoff J, Gray G, Vandebosch A (2021). Safety and efficacy of single-dose Ad26.COV2.S vaccine against Covid-19. N Engl J Med.

[10] Nishizawa Y, Hoshina Y, Baker V (2021). Bell's palsy following the Ad26.COV2.S COVID-19 vaccination [In Press]. QJM.

[11] Soeiro T, Salvo F, Pariente A, Grandvuillemin A, Jonville-Béra AP, Micallef J (2021). Type I interferons as the potential mechanism linking mRNA COVID-19 vaccines to Bell's palsy [In Press]. Therapie.

